# Injury Surveillance in Early Specialized Adolescent Competitive Swimmers: A 4-Year Prospective Study: Corrigendum

**DOI:** 10.1249/ESM.0000000000000069

**Published:** 2026-07-22

**Authors:** Takao Mise, Miwako Suzuki-Yamanaka, Yuiko Matsuura

Following publication of the article titled “Injury Surveillance in Early Specialized Adolescent Competitive Swimmers: A 4-Year Prospective Study,” published in May 2026 in *Exercise, Sport, and Movement*, an error in the exposure calculations was discovered.

To correct the error, the incidence rates per 1000 athlete hours have been updated throughout the paper, including in Tables 2, 3, and 4 (see below).

**Table 2:** Participant Exposure, Number of Injuries, and Incidence Rate.

	Exposure (AH)	Number of Injuries	Incidence Rate Per 1000 AH (95% CI)	Incidence Rate Ratio (95% CI)
All	127,929.75	111	0.868 (0.706–1.029)	1.20 (0.90–1.61)
Males	55,973.25	36	0.643 (0.433–0.853)	(ref)
Females	71,956.50	75	1.042 (0.806–1.278)	**1.62 (1.09–2.41**)

Bold values indicate a statistically significant difference.

(ref), reference value; AH, athlete hours; CI, confidence interval.

**Table 3:** Numbers of Injuries and Incidence Rates by Injury Severity, Injured Body Part, and History of Injury.

Category (Exposure)	Factor		Number of Injuries	Incidence Rate Per 1000 AH (95% CI)		Incidence Rate Ratio (95% CI)
All (127,929.75 AH)		Injury severity				
		Time loss	27	0.211	(0.131–0.291)	(ref)
		Non-time loss	84	0.656	(0.516–0.797)	**3.11 (2.02–4.80**)
		Injured body part				
		Shoulder	44	0.344	(0.242–0.446)	(ref)
		Lumbar	19	0.149	(0.082–0.215)	**0.43 (0.21–0.87**)
		Knee	14	0.109	(0.052–0.167)	**0.32 (0.15–0.68**)
		Ankle	34	0.266	(0.176–0.355)	0.77 (0.41–1.46)
		History of Injury				
		New	71	0.555	(0.426–0.684)	**1.78 (1.20–2.62**)
		Recurrent	40	0.313	(0.216–0.410)	(ref)
Male (55,973.25 AH)		Injury severity				
		Time loss	7	0.125	(0.032–0.218)	(ref)
		Non-time loss	29	0.518	(0.330–0.707)	**3.11 (2.02–4.80**)
		Injured body part				
		Shoulder	13	0.232	(0.106–0.359)	(ref)
		Lumbar	5	0.089	(0.011–0.168)	
		Knee	3	0.054	(−0.007 to 0.114)	
		Ankle	15	0.268	(0.132–0.404)	1.15 (0.55–2.42)
		History of injury				
		New	29	0.518	(0.330–0.707)	**4.14 (1.81–9.46**)
		Recurrent	7	0.125	(0.032–0.218)	(ref)
Female (71,956.50 AH)		Injury severity				
		Time loss	20	0.278	(0.156–0.400)	(ref)
		Non-time loss	55	0.764	(0.562–0.966)	**2.75 (1.65–4.59**)
		Injured body part				
		Shoulder	31	0.431	(0.279–0.582)	(ref)
		Lumbar	14	0.195	(0.093–0.296)	**0.45 (0.24–0.85**)
		Knee	11	0.153	(0.063–0.243)	**0.35 (0.18–0.71**)
		Ankle	19	0.264	(0.145–0.383)	0.61 (0.35**–**1.08)
		History of injury				
		New	42	0.584	(0.407–0.760)	**1.27 (0.81–2.01**)
		Recurrent	33	0.459	(0.302–0.615)	(ref)

Bold values indicate a statistically significant difference.

(ref), reference value; AH, athlete hours; CI, confidence interval.

**Table 4:** Incidence Rates of Injured Body Parts, Injury Types, Modes of Onset, Severity, and History of Injuries by Sex.

	Incidence Rate Per 1000 AH (*n*)		Incidence Rate Ratio (95% CI)
	Males (55,973.25 AH)	Females (71,956.50 AH)	
Body part			
Shoulder	0.232 (13)	0.431 (31)	1.85 (0.97–3.54)
Lumbar	0.089 (5)	0.195 (14)	-
Knee	0.054 (3)	0.153 (11)	-
Ankle	0.268 (15)	0.264 (19)	0.99 (0.50–1.94)
Injury type			
Impingement syndrome	0.125 (7)	0.125 (9)	1.00 (0.37–2.69)
Tendinopathy	0.125 (7)	0.111 (8)	0.89 (0.32–2.45)
Unknown, or not specified	0.107 (6)	0.083 (6)	0.78 (0.25–2.41)
Arthritis	0.089 (5)	0.083 (6)	-
Muscle enthesopathy	- (2)	0.111 (8)	-
Joint sprain/ligament tear	- (0)	- (2)	-
Bone stress injury	- (0)	- (1)	-
Osteochondrosis	- (0)	- (1)	-
Bursitis	- (0)	- (1)	-
Mode of onset			
Repetitive, gradual	0.465 (26)	0.514 (37)	1.11 (0.67–1.83)
Repetitive, sudden	- (1)	- (4)	-
Acute, sudden	- (2)	- (1)	-
Severity			
Time loss	0.125 (7)	0.278 (20)	2.22 (0.94–5.26)
Non-time loss	0.518 (29)	0.764 (55)	1.48 (0.94–2.31)
History of injury			
New	0.518 (29)	0.584 (42)	1.13 (0.70–1.81)
Recurrent	0.125 (7)	0.459 (33)	**3.67 (1.62–8.29**)

Bold values indicate a statistically significant difference.

AH, athlete hours; CI, confidence interval.
